# Anti-Yo and anti-glutamic acid decarboxylase antibodies presenting in carcinoma of the uterus with paraneoplastic cerebellar degeneration: a case report

**DOI:** 10.1186/1752-1947-6-155

**Published:** 2012-06-13

**Authors:** Peter K Panegyres, Angela Graves

**Affiliations:** 1 Neurodegenerative Disorders Research Pty Ltd, 185 York Street, Subiaco, 6009, Western Australia; 2 Neurology Service, Joondalup Health Campus, Joondalup, Perth, Western Australia

**Keywords:** 14-3-3 proteins, anti-Yo/anti-GAD antibodies, clear cell carcinoma of uterus, paraneoplastic cerebellar degeneration

## Abstract

**Introduction:**

Paraneoplastic cerebellar degeneration is a rare non-metastatic manifestation of malignancy. In this report, to the best of our knowledge we describe for the first time a diagnosis of paraneoplastic cerebellar degeneration several months prior to the diagnosis of clear carcinoma of the uterus.

**Case presentation:**

A 75-year-old Caucasian woman manifested a rapidly progressive cerebellar syndrome with nystagmus, past-pointing, dysdiadochokinesis, dysarthria, truncal ataxia and titubation. The paraneoplastic cerebellar degeneration was associated with anti-Yo and anti-glutamic acid decarboxylase antibodies. 14-3-3 protein was detected in the cerebrospinal fluid. She was treated with intravenous immunoglobulin prior to laparotomy, hysterectomy and bilateral salpingoophorectomy. Our patient has survived for three years following diagnosis and treatment.

**Conclusions:**

To the best of our knowledge this is the first report of an association of clear cell carcinoma of the uterus and paraneoplastic cerebellar degeneration with both anti-Yo and anti-glutamic acid decarboxylase antibodies. The findings imply that both antibodies contributed to the fulminating paraneoplastic cerebellar degeneration observed in our patient, and this was of such severity it resulted in the release of 14-3-3 protein in the cerebrospinal fluid, a marker of neuronal death.

## Introduction

Adult onset progressive cerebellar degenerations represent complex diagnostic and management challenges for which genetic and non-genetic causes are recognized. The non-genetic causes are alcohol and toxins, immune mediated, vitamin deficiency, leptomeningeal deposition such as superficial siderosis and chronic infections such as Whipple’s disease [1,2]. Prion disorders and mutations in single genes might also cause sporadic ataxia in adults.

Paraneoplastic cerebellar degeneration (PCD) is one of the most important causes of immune-mediated degenerative cerebellar pathology. This is an uncommon condition and can be associated with many different types of tumor, including small cell cancer of the lung, cancer of the breast or ovary and lymphoma [3].

Of the gynecological causes, cancers of the ovary are well recognized. Less appreciated are cancers of the endometrium. In this report, to the best of our knowledge we describe for the first time the association of clear cell endometrial carcinoma with PCD, anti-Yo, and anti-glutamic acid decarboxylase (GAD) antibodies with detectable protein 14-3-3 in the cerebrospinal fluid (CSF). The diagnosis of PCD led to the diagnosis of endometrial carcinoma.

## Case presentation

A 75-year-old Caucasian woman reported a three-month period of general constitutional decline with the onset of vertiginous symptoms and vomiting one week prior to attending our emergency department. She had been living independently. Her medical history included paroxysmal atrial fibrillation treated with amiodarone and warfarin, hypertension controlled with metoprolol and irbesartan/hydrochlorothiazide, and right knee osteoarthritis. She does not have diabetes mellitus. Her initial neurological examination results were unremarkable. She received a putative diagnosis of viral labyrinthitis and was admitted for intravenous fluid replacement. Over the next week she experienced troublesome vertigo but was able to be mobile with use of a Zimmer frame. Her course was complicated one week after admission by a fracture of the lateral mass of C2 after falling whilst mobilizing to the toilet. This required hard-collar immobilization, which made further investigation problematic. At this stage she manifested a clinical rapidly progressive cerebellar syndrome with nystagmus, past-pointing, dysdiadochokinesis, dysarthria, truncal ataxia and titubation. Her functional status deteriorated to a modified Rankin score of 5.

A brain MRI scan revealed no structural abnormalities. Anti-Yo antibodies were found to be present in serum and CSF, as were anti-GAD antibodies (Table 1). The CSF showed 0 leukocytes/μL and 50 erythrocytes/μL, gave negative results on Gram stain and negative results on culture, her protein level was 0.44 g/L and glucose level 3.2 mmol/L (concurrent blood glucose level 5.6 mmol/L), and cytology results were negative for malignant cells. Oligoclonal bands were not detected. Her CSF was also positive for protein 14-3-3, with a single band identified on western blot immunodetection. Ca 15–3 and Ca 125 titers were also mildly elevated in her blood.

**Table 1:** Paraneoplastic cerebellar degeneration

**Date**	**Fluid**	**Antibodies**		**Proteins**	**Findings**
		**Anti-Yo**	**Anti-GAD**		
1 August 2008	Serum	2U (range 0 to 3)	4.3U/mL (<1)		Anti-Yo confirmed on immunoblot
	CSF	1U	2.0U/mL	14-3-3	
		Serum:CSF ratio = 2:1	Serum:CSF ratio = 2.15:1		
5 August 2008		Intravenous Ig 28 g (0.4 g/kg per day for five days)			
1 September 2008		Laparotomy, hysterectomy, bilateral salpingoophorectomy			
9 April 2009	Serum	1U	0.2U/mL		
	CSF	Undetectable	Undetectable		
23 December 2009	Serum	0	<0.1U/mL		
24 December 2010	Serum	0	<0.1U/mL		
7 November 2011					Patient alive; Rankin score 5

Anti-Yo antibodies detected by indirect immunofluorescence on a composite block of primate cerebellum and cerebrum, and mouse stomach (titers expressed 0 = not detected; 1 = weak positive; 2 = moderate positive; 3 = strong positive). GAD antibodies (GAD 65) are measured using a radioimmunoassay and expressed in U/mL (reference range <1).

CSF, cerebrospinal fluid; GAD, glutamic acid decarboxylase.

A positron emission tomography (PET) scan revealed a fludeoxyglucose-avid bulky lower uterus evident with diffuse cerebellar hypo-activity. Pelvic ultrasound revealed a 4 × 3 cm uterine cyst. A hysterectomy and bilateral salpingoophorectomy confirmed a clear cell carcinoma of the endometrium. A diagnosis of stage II endometrial carcinoma involving and extending into the stroma of the cervix with vascular invasion and histological grade 3 was made. She received five cycles of intravenous immunoglobulin (Ig) prior to surgery. The original C2 spinal injury and multiple infective complications including aspiration pneumonia, line sepsis, percutaneous endoscopic gastrostomy (PEG) site infection and T8/9 discitis made her management difficult. Five months elapsed from presentation to diagnosis of her neoplasm. She was too frail for adjuvant chemotherapy. She was discharged to nursing home care in April 2009, where she remains to the present day.

She was assessed in December 2009 in her nursing home where she was found to be bright and alert. She was dysarthric. She was able to engage in light-hearted conversation in relation to the performance of Australian Rules football teams in English and Macedonian. She was mostly feeding by mouth. The PEG feed was rarely used. She required a hoist for transfers. She was able to place both hands against gravity under command. There was mild tremor of her outstretched hands. There was impaired finger-to-nose testing that had improved from the examination of 21 April 2009. She was able to raise both legs against gravity. There was marked dysmetria in both lower limbs, again much less than as an in-patient. Deep tendon reflexes were 1+. There was no clonus. She had nystagmus in lateral gaze without gaze paresis. There was some limitation in her upward gaze. Convergence and accommodation reaction were normal. The cranial nerve examination was otherwise normal.

When reassessed in August 2011 she remained bed bound without changes in the neurological examination. She remains alive to this day, dependent on staff for all acts of daily living and requiring feeding assistance. Her weight ranges from 52.8 kg to 61.8 kg.

## Discussion

PCD is rare and not commonly associated with endometrial cell carcinoma, particularly clear cell endometrial carcinoma [4]. PCDs have however been associated with fallopian tube adenocarcinoma [5]. To the best of our knowledge this is the first case of clear cell endometrial carcinoma with anti-Yo antibodies in serum and CSF, associated with anti-GAD antibodies in serum and CSF; observations that extend the known associations and relationships of both of these antibodies and indicates the importance of an aberrant immune response in PCD. Our patient’s case is also unique in that 14-3-3 proteins were identified in the CSF, probably related to neuronal death. Furthermore our patient was given intravenous immunoglobulin and had surgical treatment for the endometrial carcinoma. She has remained alive though disabled for over three years. This therapeutic outcome supports the notion of a poor therapeutic response to intravenous immunoglobulin in patients with anti-Yo associated PCDs [6] and not a positive treatment effect observed by others [7,8].

The titers of anti-Yo and anti-GAD antibodies in our patient decreased in both blood and CSF following treatment, reflecting the reduced immunological response to the tumor provided by its surgical extirpation and treatment with intravenous Ig (Table 1). Our patient’s case highlights that the presence of the 14-3-3 protein in CSF, even though having high sensitivity and specificity for prion diseases may also be found with PCDs, including non-clear cell carcinoma of the uterus [9]. This is an important practice point in that in a patient with a cerebellar syndrome, where there might be considerations of a prion disease and where 14-3-3 proteins are found, the physician needs to consider the possibility of an underlying cancer.

14-3-3 proteins have previously been observed in a patient with anti-GAD associated cerebellar ataxia [10]. Furthermore anti-GAD antibodies are not only associated with stiff-person syndrome but in about 20% with cerebellar ataxia [11]. Our patient is distinctive in that both the anti-Yo and anti-GAD antibodies can be implicated in the cerebellar degeneration.

The pathophysiology of the cerebellar degeneration is probably a consequence of a direct cytotoxic effect of the anti-GAD antibodies on the Purkinje cells [12] and possibly an apoptotic effect of the anti-Yo antibody after uptake by cerebellar Purkinje cells [13-15]. The pathophysiological effects of both antibodies resulted in the fulminating and devastating cerebellar syndrome in our patient. The initial cell damage might involve cell-mediated immunity, as these antibodies recognize intracellular antigens; this mechanism is supported by the unsuccessful attempts to transfer the disease in animals by antibodies or immunization.

## Conclusions

To the best of our knowledge this is the first report of a PCD caused by clear cell carcinoma of the uterus with both anti-Yo and anti-GAD antibodies, in association with 14-3-3 proteins in CSF. These observations increase our understanding of the antibodies related to PCD in gynecological malignancies and is of relevance to neurologists, gynecologists, general internists, oncologists and clinical immunologists.

## Consent

Written informed consent was obtained from the patient for publication of this case report. A copy of the written consent is available for review by the Editor-in-Chief of this journal.

## Competing interests

The authors declare that they have no competing interests.

## Authors’ contributions

PKP and AG were both responsible for diagnosis and patient management, and both wrote and approved the final manuscript.
